# Generic orphan drug substitution: a critical analysis of global practices and Saudi Arabia’s perspective

**DOI:** 10.3389/fphar.2024.1376009

**Published:** 2024-04-18

**Authors:** Yousif S. Alakeel, Emmanouil Rampakakis, Ali AlRumaih, Rana AlRuwaisan, Maha Abushal, Abdullah M. AlDalaan, Majdy M. Idrees, Zaid D. Alanazi, Hanouf AlKoait, Abdulrahman Muaadi, Majed Ali M. AlAfra, Shaya A. AlShaya, Suliman AlHomida

**Affiliations:** ^1^ College of Pharmacy, King Saud Bin Abdulaziz University for Health Sciences, Riyadh, Saudi Arabia; ^2^ Department of Pharmaceutical Care Services, King Abdulaziz Medical City, Riyadh, Saudi Arabia; ^3^ JSS Medical Research Inc., Scientific Affairs, Montreal, QC, Canada; ^4^ Department of Pediatrics, McGill University, Montreal, QC, Canada; ^5^ General Directorate for Health Services at Ministry of Defense, Riyadh, Saudi Arabia; ^6^ King Fahad Medical City, Riyadh, Saudi Arabia; ^7^ Prince Sultan Military Medical City, Riyadh, Saudi Arabia; ^8^ King Faisal Specialist Hospital and Research Center, Riyadh, Saudi Arabia; ^9^ King Saud University Medical City, Riyadh, Saudi Arabia; ^10^ King Khaled University Hospital, Riyadh, Saudi Arabia

**Keywords:** generic, biosimilar, orphan, rare, Middle East

## Abstract

In an era of cost pressure, substituting generic drugs represents one of the main cost-containment strategies of healthcare systems. Despite the obvious financial benefits, in a minority of cases, substitution may require caution or even be contraindicated. In most jurisdictions, to obtain approval, the bioequivalence of generic products with the brand-name equivalent needs to be shown via bioavailability studies in healthy subjects. Rare diseases, defined as medical conditions with a low prevalence, are a group of heterogenous diseases that are typically severe, disabling, progressive, degenerative, and life-threatening or chronically debilitating, and disproportionally affect the very young and elderly. Despite these unique features of rare diseases, generic bioequivalence studies are typically carried out with single doses and exclude children or the elderly. Furthermore, the excipients and manufacturing processes for generic/biosimilar products can differ from the brand products which may affect the shelf-life of the product, its appearance, smell, taste, bioavailability, safety and potency. This may result in approval of generics/biosimilars which are not bioequivalent/comparable in their target population or that meet bioequivalence but not therapeutic equivalence criteria. Another concern relates to the interchangeability of generics and biosimilars which cannot be guaranteed due to the phenomenon of biocreep. This review summarizes potential concerns with generic substitution of orphan drugs and discusses potentially problematic cases including narrow therapeutic index drugs or critical conditions where therapeutic failure could lead to serious complications or even death. Finally, we put forward the need for refining regulatory frameworks, with emphasis on Saudi Arabia, for generic substitution and recent efforts toward this direction.

## 1 Introduction

### 1.1 Rare diseases

Rare diseases are defined as medical conditions with a low prevalence. Definitions for rare diseases differ across jurisdictions and no single definition is accepted worldwide (Loorand-Stiver et al., 2016).

In Saudi Arabia (Saudi Food and Drug Authority, 2023), rare disease is defined as any life-threatening, seriously debilitating or serious and chronic condition which:

a) affects fewer than five in 10,000 individuals.

OR

b) affects more than five in 10,000 individuals but for which there is no reasonable expectation that the cost of developing and making available in Saudi Arabia a drug for such disease or condition will be recovered from sales of such drug.

Rare diseases are a group of heterogenous diseases that, in addition to their scarcity, share important characteristics: they are typically severe, disabling, progressive, degenerative, and life-threatening or chronically debilitating (Flather et al., 1997; Institute of Medic ine Committee on Accelerating Rare Diseases R et al., 2010). Approximately two-thirds of rare diseases have their onset in childhood and the majority (70%–80%) are genetic in origin, affecting between 3% and 4% of births, or have a genetic component (Flather et al., 1997; Thorat et al., 2012; Zhao et al., 2014; Bu et al., 2020). A large percentage of rare diseases are cancers; however, rare diseases span all therapeutic areas.

With an estimated number of rare diseases of approximately 7,000, the number of individuals affected by a rare condition is collectively large and estimated at approximately 10% of the global population (Zhao et al., 2014). The MENA region has one of the highest prevalence rates in the world for rare diseases, particularly those with a genetic component; mostly due to large family sizes, advanced maternal/paternal age, and a high rate of consanguineous marriages (Almalki et al., 2012). With a combined population of approximately 400 million, the estimated prevalence of rare diseases in the MENA region is three million patients, with Egypt (∼620,000) and Saudi Arabia (∼320,000) being the major contributors. The most common rare diseases in the region include hemoglobinopathies (e.g., sickle cell anemia and thalassemia), glucose-6-phosphate dehydrogenase deficiency, autosomal recessive syndromes (e.g., cystic fibrosis and spinal muscular atrophy), certain inborn errors of metabolism (e.g., Pompe’s disease, Mucopolysaccharidosis types), Behcet’s disease, and several metabolic disorders (e.g., Gaucher and Fabry disease).

Treatment for rare diseases tends to be expensive and complicated, involving high medical needs, long stays in hospital, work loss, early retirement, requirement of caregiver assistance, and non-medical costs associated with necessary modification of homes or vehicles. Thus, it represents a growing burden for governments and health authorities, including those in the MENA region. In a recent initiative of the EveryLife Foundation, a comprehensive evaluation of the economic burden of all rare diseases pooled together in the US was undertaken, including costs often not considered in cost effectiveness assessments due to non-availability (Yang et al., 2022). Results of this evaluation showed that the estimated total economic burden of rare diseases in the US reached nearly $1 trillion in 2019, with indirect and non-medical costs representing the majority (57%) of the cost. Despite the emphasis often placed on the cost of prescription medications, these accounted only for 5% of the total burden. More recently, another study estimated the cost of rare diseases in the US to be $2.2 trillion per year and identified mortality cost as another key driver of economic burden (Andreu et al., 2022).

### 1.2 Orphan drugs for rare diseases

Orphan drugs are therapeutic agents used for the management of rare diseases. In the last decade, there has been a shift in the research and development priorities of the pharmaceutical industry from blockbuster to niche drugs which is reflected in the increasing number of orphan drug approvals over time and the rate of increase in the last few years (Yang et al., 2022). In a 2022 comparative analysis of the orphan drug policies in Saudi Arabia, the US, and the EU, it was shown that among 619 products across 792 indications, the FDA had approved 594, EMA approved 329, and SFDA registered 171 (Balkhi et al., 2023).

### 1.3 Generic substitution

Generics are pharmaceutically equivalent to the reference products, being medicines with the same qualitative and quantitative composition in the active ingredient(s), dosage form, strength, and route of administration. However, they may differ in terms of excipients or additional constituents which potentially affect drug stability, absorption, and toxicity (Tassi et al., 2017).

Generic substitution refers to the replacement of a branded medical product by a generic version. As generic drugs are typically less expensive than the innovator product, their use is encouraged or, in certain cases, obligated by health authorities across the world to reduce healthcare spending. Despite the obvious financial benefits and evidence that generic substitution can be free of major complications, orphan drug substitution may require caution in some cases or even be contraindicated.

#### 1.3.1 Definition of bioequivalence

In most countries, to obtain approval, manufacturers which develop generics need to demonstrate the product bioequivalence with the brand-name equivalent by appropriate bioavailability studies in healthy subjects (Gozzo et al., 2022). The parameters used to measure bioavailability include the area under the plasma concentration–time curve (AUC) and the maximal plasma concentration (Cmax). A product can be considered bioequivalent to the brand-name drug if after administration of the same dose, it exhibits a similar degree and rate of absorption. Average bioequivalence is established if the 90% confidence interval (CI) for the geometric mean of both the AUC and Cmax for the generic product are within 80% and 125% of the corresponding parameters for the innovator product (Heaney and Sander, 2007; Baumgärtel, 2012). For drugs with narrow therapeutic index (NTI), i.e., drugs where small differences in blood concentration may lead to serious therapeutic failures or adverse drug reactions, many agencies recommend more stringent limits (90% CI: 90%–111%) (Gozzo et al., 2022).

### 1.4 Biosimilars

A biosimilar is a biological medicine that is highly similar to an existing (“reference”) biologic. Through a comprehensive comparability exercise, manufacturers of biosimilars are required to demonstrate that their product is highly similar to the reference medicine, notwithstanding natural variability inherent to all biological medicines, and there are no clinically meaningful differences in terms of safety, purity, and potency. This comparability exercise can include quality/analytical data, *in vitro* and *in vivo* non-clinical data, and clinical data to demonstrate biosimilarity in terms of structure, function, toxicity, pharmacokinetics, pharmacodynamics, clinical immunogenicity, safety and efficacy (FDA, 2015; EMA, 2019). Assessment of biosimilarity typically follows a step-wise process that is tailor-made for each product. Decisions are typically based on the totality of evidence submitted and agencies have the discretion to determine that an element described above is unnecessary based on a risk-based approach.

## 2 Challenges

Although issues with generic substitution of specific medication classes have been reported (e.g., post-transplantation immunosuppressants, anti-epileptic drugs, and antidepressants), very limited data are currently available on the impact of generic substitution of orphan drugs due to the small number of patients. One such example of problematic generic substitution in rare diseases involves the generic substitution of levothyroxine in children with severe congenital hypothyroidism (Carswell et al., 2013; Bate et al., 2016a). Based on the available literature, additional concerns with substitution of several other orphan medications (e.g., fosphenytoin for the treatment of generalized convulsive status epilepticus) exist; however, they remain to be proven/disproven (Di Paolo and Arrigoni, 2018; Elmer and Reddy, 2022).

### 2.1 Differences in product formulations and manufacturing: bioequivalence vs. therapeutic equivalence

The excipients and manufacturing processes for generic products can differ from the brand products, which may affect the stability of the product (i.e., its shelf life) or its chemical form in terms of salt or ester of the active ingredient (Davies, 2001; Verbeeck et al., 2006). Though these molecules are often considered inactive or inert, the former may affect the stability of the active ingredient and the latter can alter the chemical and biological properties of the active ingredient (Borgheini, 2003; Genazzani and Pattarino, 2008). Furthermore, there may be differences in the appearance, smell, taste, and shelf life between generics and with the branded drugs (Guberman and Corman, 2000). As a result, generic switching may interfere with patient adherence to treatment, particularly in children, be associated with suboptimal clinical and safety outcomes, or even result in additional healthcare resource utilization and costs of care (Meredith, 2003; Thiebaud et al., 2005; Verbeeck et al., 2006; Ansell, 2008; Genazzani and Pattarino, 2008; Bainbridge and Ruscin, 2009; Håkonsen et al., 2009; Desmarais et al., 2011; Straka et al., 2017).

In most therapeutic areas, the aforementioned differences have negligible impact on the drugs’ pharmacological activity and, thus, equivalent bioavailability translates to therapeutic equivalence. However, in certain cases, particularly in complex diseases and complex treatment regimens, generic substitution has been associated with decreased treatment efficacy and tolerability issues and careful evaluation is required (Bate et al., 2016b; Straka et al., 2017).

Given the aforementioned differences and considering that efficacy studies are not necessary for the approval of generics, there are concerns about the therapeutic equivalence of generic drugs. These concerns are reinforced in the case of orphan drugs, for which appropriately sized post-approval studies for each generic are close to impossible given the scarcity of the patient populations. The lack of therapeutic equivalence has further implications in rare diseases due to the fact that they disproportionally affect the pediatric population. Specifically, there are concerns associated with the fragile nature of neonates, infants, and toddlers and the occurrence of adverse events, the potentially irreversible and/or critical effects that suboptimal drug performance may have, as well as a potentially missed a therapeutic opportunity due to switch to a subsequent line of treatment instead of maximizing benefits from the originator/reference drug prior to proceeding to subsequent options.

### 2.2 Differences between healthy subjects and target patient populations

A key criticism of generic substitution is that studies evaluating the relative bioavailability of generic and brand orphan drugs are performed in a homogeneous group of healthy volunteers aged 18–55 years who do not take concurrent medications and maintain an overall healthy lifestyle, and not the patient population for which the drug is approved.

As a result, bioequivalence studies do not consider known variations in pharmacokinetics associated with age or disease particularities (Kearns et al., 2003; Meredith, 2003; Crawford et al., 2006; Straka et al., 2017). Although bioequivalence studies use a crossover design to account for intrasubject variability, they are not designed to detect differences in the pharmacokinetics of generic and brand medications that may exist in excluded age groups such as children or the elderly. This is even though more than two-thirds of orphan disease patients fall within the tails of age distribution (Flather et al., 1997; de Barros and Papoila, 2007; Bu et al., 2020). Furthermore, bioequivalence studies do not consider potential differences in the bioavailability of generic and originator products, which may exist in patients with concurrent diseases and medications due to first-pass metabolism, or due to the influence of fasting status.

Therefore, in patients and specific patient subgroups with pharmacokinetic parameters that may vary compared to healthy individuals, caution is warranted when considering generic substitution because the two formulations may be inequivalent in terms of bioavailability (Sabatini et al., 1999; Meredith, 2003).

### 2.3 Extrapolation of single-dose bioequivalence to chronic use

Bioequivalence studies are usually carried out with single doses. The use of single-dose studies to predict the results of multiple-dose use is another concern (Meredith, 2003) with generic substitution. In its guidance for industry regarding bioavailability and bioequivalence studies, the FDA recommends single-dose studies because “*they are generally more sensitive than steady-state studies in assessing rate and extent of release of the drug substance from the product into the systemic circulation”* (Hedges and Olkin, 2014). However, some studies have shown that differences in the rate and extent of absorption of enteric-coated products become more pronounced after multiple doses (Elkoshi et al., 2002), suggesting that differences in bioavailability may be missed by bioequivalence studies. This is particularly relevant as therapeutic benefit in chronic conditions is dependent on the maintenance of steady state and does not only rely on single-dose pharmacokinetics. Furthermore, it has been suggested that the presence of apparently inert compounds in generics which are absent from the brand drugs, discussed above, could affect long-term, but not single-dose, distribution, absorption, or metabolism (Besag, 2000). The impact of such variations in bioavailability would be more pronounced in drugs with NTI, and could lead to serious adverse reactions or therapeutic failure (Jiang et al., 2015).

Therefore, at least in some cases, multiple-dose studies would be recommended for evaluation of bioequivalence.

### 2.4 Interchangeability of generics

In different jurisdictions, medical doctors are obligated to fill the prescription only with the active substance of the product and the pharmacists may automatically substitute generic and brand products as well as between generics, depending on price and availability, which can lead to frequent switching between formulations.

**FIGURE 1:**
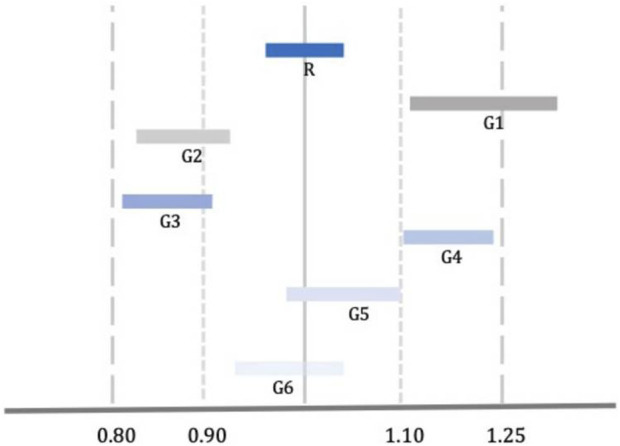
Comparison of Bioequivalence of Reference (R) and Generic (G) Drugs. G3 and G4 meet the standard criteria of bioequivalence; however, they are not bioequivalent to each other [Adopted from Gozzo et al. (2022)].

Although the approval of a generic is based on its bioequivalence with the brand product, the bioequivalence of different generic versions is not guaranteed due to the “biocreep” phenomenon (Gozzo et al., 2022). One such example is shown in Figure 1, where two generics (G3 and G4) meet the bioequivalence criteria against the reference product but lay at the lower and upper borders of the bioequivalence interval which makes them bio-inequivalent. Switching from G3 to G4 without dose adjustment would result in significant reduction in plasma drug levels with potential loss of efficacy. Switching from G4 to G3 without dose adjustment would result in an increase of plasma drug levels with possible adverse drug reactions. Concerns about the “biocreep” phenomenon apply to all drugs and therapeutic areas; however, they are pronounced (a) in drugs with NRI, and (b) in cases where patients are stabilized following dose titration and switch to a different version of the product. In such cases, switch of generics may result in loss of control which, in turn, could be fatal (e.g., epilepsy, pulmonary hypertension, etc.).

### 2.5 Complex drugs

Among others (e.g., products that have complex active ingredients, complex drug-device combination products), complex drugs include those that are intended to deliver medication to local sites of action, such as the lung (e.g., inhaled or nebulized medications for respiratory disease) or the skin (e.g., dermatological creams, gels, ointments, or patches). Demonstrating bioequivalence of these types of medications is associated with unique challenges due to difficulties in measuring the rate of absorption as well as variations in excipients (e.g., particle size for inhalers, delivery systems for topical medications) which can significantly influence medication release, absorption, stability, and ultimately, the therapeutic effect, of two drugs with the same active ingredient and dose (Williams and Barry, 2012; Xu et al., 2014; Hmingthansanga et al., 2022).

Despite these challenges, most government regulatory agencies demand that the generic complex products prove both pharmaceutical equivalence and bioequivalence via pharmacokinetic and clinical endpoint or pharmacodynamic studies. Although comparative clinical studies are still considered the gold standard approach for establishing bioequivalence in most formulations, these studies can be costly and insensitive to detect formulation differences. Thus, demonstrating bioequivalence of complex drugs often requires alternative *in vitro* and *in vivo* methods, advanced analytical technologies, quantitative methods, and modeling and data analytics methodologies to establish scientific standards that would ensure therapeutic equivalence in patients (Zhang et al., 2023; Alomari and Alhussaini, 2024). Further refinement and standardization of these novel methodologies for regulatory purposes and product-specific guidances are needed to support the development and approval of safe and effective complex generic drugs (FDA, 2024).

### 2.6 Biosimilarity and interchangeability

Demonstrating biosimilarity of a subsequent entry product to the reference product requires extensive comparability studies (Section 2.4). Due to the low prevalence of rare diseases, limited clinical data are available for biosimilars, which may raise concerns about the effectiveness and safety of these products (Allocati et al., 2022). Therefore, establishment of a robust pharmacovigilance system to ensure monitoring of the safety and patient experience with biosimilars once they are on the market is critical (Oza et al., 2019; Drelichman et al., 2020).

Another particularity with biosimilars relates to their interchangeability, i.e., the potential to be substituted by another biosimilar of the same reference product without consulting the prescribing healthcare professional, often referred to as “pharmacy-level substitution”. Once a biosimilar is approved, some agencies (e.g., WHO, EMA) automatically consider them interchangeable while others (e.g., FDA) differentiate between biosimilarity and interchangeability with additional data required for granting the latter classification (Drelichman et al., 2020).

There is a lack of extensive data on the post-approval comparability of biosimilars for rare diseases. However, a recent analysis of the original imiglucerate product, an enzyme replacement therapy used to treat Gaucher disease, and two subsequent products that were not approved via biosimilar regulatory pathways showed considerable differences in the safety profile (Tsang et al., 2022). Although these findings are not necessarily representative of approved biosimilars, they do highlight the need for a stringent safety reporting system, clear regulations on the safety reporting requirements, as well as the collection of long-term real-world effectiveness data (Drelichman et al., 2020).

## 3 Overview of current health authorities’ practices

In recognition of the fact that the use of a single regulatory acceptance range for all drugs is problematic, both the FDA and the EMA previously launched programs with product-specific guidance (PSG) for generic drug development which advocate that the selection of the method used to demonstrate bioequivalence should depend upon the purpose of the study, the analytical methods available, and the nature of the drug product. Furthermore, to increase transparency on PSG and the FDA’s current best thinking on the topic and also to ensure that policies and regulations keep pace with the evolving science of equivalence, the FDA recently started publishing on a regular basis and in a timely fashion upcoming new and revised PSGs as a commitment under the Generic Drug User Fee Amendments of 2022 (GDUFA III) (FDA, 2024). No revisions to the Saudi Food and Drug Authority’s Guidelines for Bioequivalence or the Harmonised Arab Guideline on Bioequivalence of Generic Pharmaceutical Products (endorsed by health regulatory authorities’ representatives from the MENA region) have been published since 2011 and 2014, respectively.

In terms of generic substitution, legislation differs considerably among countries; a few examples being presented below. In the US, less than half (n = 19) of the states mandate generic substitution by pharmacists when generic products are available; the remaining 31 states permit but do not require substitution (Sacks et al., 2021). Furthermore, seven states require that patients consent to substitution, while in 23 states patients maintain the right to refuse substitution without requiring that they consent. UK law does not permit generic substitution, except in emergency cases (Department of Health and Social Care, 2021). While in Italy, physicians should fill in the prescription by indicating the name of the active substance if the patient is being treated for the first time for a chronic disease or is being treated for a new episode of a non-chronic disease if generics of the product are available. However, they can prescribe a specific product if it is considered not replaceable for a specific patient, but the non-replaceability clause must be necessarily and properly justified (Gozzo et al., 2022). No specific guidance is available for generic substitution in Saudi Arabia or the regions of Middle East and Northern Africa (MENA) and the Gulf Cooperation Council (GCC). Irrespective of the above variation between countries, the absence of consideration to the biocreep phenomenon and the interchangeability of various generics is a common denominator of all frameworks to date. Guidelines for biosimilar development were published by the WHO in 2009, the FDA, the EMA and more recently in Japan, Canada and Australia. However, guideline adoption in regions such as the MENA, Latin America and Asia remains slow.

## 4 Discussion

### 4.1 Closing remarks and way forward

Although very limited data are currently available on the impact of generic substitution of orphan drugs, cumulative evidence from complex diseases in the literature suggests that, in some cases, the interchangeability of branded and generic products is not guaranteed, and caution should be exercised in treatment selection. This is particularly important for conditions where relatively small variations in bioavailability may have major impact on their efficacy or tolerability, critical conditions where therapeutic failure could lead to serious complications or even death, drugs with complex dosage regimens, and conditions treated with polymedication. To further complicate matters, it is hard to assess the consequences of substituting either brand or generic drug products in patients with progressive rare conditions because it is not clear if disease progression is due to the natural course of the condition or due to drug substitution.

There is a need for cooperation and collaboration between all stakeholders in rare disease and orphan drug research and development to come up with a clear regulatory framework for generic substitution in Saudi Arabia and the MENA/GCC regions taking into consideration the particularities of different therapeutic areas, rare diseases, and specific drugs. These guidelines need to consider the critical phenomenon of biocreep and be updated on a regular basis to reflect current scientific knowledge and align with international counterparts.

Finally, generation of local data, reflecting systemic/cultural idiosyncrasies and taking into consideration all perspectives, is also key to accurately quantify the regional cost-of-illness of rare diseases and assess the cost-effectiveness of generics in Saudi Arabia and the MENA/GCC regions. Acknowledging the need for sufficient numbers of patients, use of multi-country registries in the region, possibly through federated approaches, would be valuable.

### 4.2 Expert panel recommendations

Specific and strict criteria should be put in place for generic drugs that can be accepted in rare diseases, particularly progressive or critical conditions with poor outcomes: (a) Produced in high-quality, qualified, and licensed manufacturing facilities; (b) In the case of foreign suppliers/manufacturers, qualification and licensing by major regulatory authorities, e.g., FDA and EMA, could be acceptable; (c) Have been used for sufficient amount of time in reputable centers in other countries with no quality-related issues, or; (d) Appropriate therapeutic equivalence studies or switching studies from brand to generic have been conducted.

Furthermore, it is our recommendation that, once treatment for rare progressive or critical conditions has begun, the decision to switch patients to generics or to switch generics should be made in consultation with the expert treating physician. In the event of drug substitution, clear policy/guidelines should be in place to grant those patients the nearest appointment to carefully monitor patient outcomes, assess therapeutic equivalence, and consider dose adjustment, if needed. The Saudi Food and Drug Authority should regularly monitor generic drug substitution issues and keep national medical associations and patients advised on developments relevant to patient care. To achieve this, there is a need for ongoing post-marketing surveillance to detect quality, safety, or therapeutic inequivalence issues with approved generic medications. Treating physicians and community pharmacists have the duty to report to appropriate regulatory authorities serious adverse drug reactions or therapeutic failures that may be related to drug substitution, as well as make the patients aware of the potential risks associated with drug substitution, educate them how to detect signs of adverse drug reactions or therapeutic failures, and encourage them to seek help in a timely fashion.

## 5 Expert panel composition

An advisory board meeting was called to discuss patient access to orphan drugs and the potential availability of generic versions, with emphasis on pulmonary arterial hypertension (PAH). Key opinion leaders representing both the government and private sectors and affiliated with diverse institutions across Saudi Arabia were invited. Those agreeing to participate formed the expert panel that met in December 2022. Panelists were clinicians from various medical fields (e.g., cardiology, pulmonology) and the pharmaceutical sector (administrative, supply chain, and clinical pharmacy). Due to extensive discussions, the expert panel convened for two additional board meetings to further explore the issue and exchange their experiences with generic medications. These discussions and insights are documented in this white paper, aimed at informing stakeholders and policymakers.

## Data Availability

The original contributions presented in the study are included in the article/Supplementary Material, further inquiries can be directed to the corresponding author.

## References

[B1] Allocati E. Godman B. Gobbi M. Garattini S. Banzi R. (2022). Switching among biosimilars: a review of clinical evidence. Front. Pharmacol. 13, 917814. 10.3389/fphar.2022.917814 36091837 PMC9449694

[B2] Almalki Z. S. Alahmari A. K. Guo J. J. Kelton C. M. (2012). Access to orphan drugs in the Middle East: challenge and perspective. Intractable Rare Dis. Res. 1 (4), 139–143. 10.5582/irdr.2012.v1.4.139 25343087 PMC4204565

[B3] Alomari N. Alhussaini W. (2024). Update on the advances and challenges in bioequivalence testing methods for complex topical generic products. Front. Pharmacol. 15, 1330712. 10.3389/fphar.2024.1330712 38389924 PMC10881717

[B4] Andreu P. Karam J. Child C. Chiesi G. Cioffi G. (2022). The burden of rare diseases: an economic evaluation. USA: CHIESI.

[B5] Ansell B. J. (2008). Not getting to goal: the clinical costs of noncompliance. J. Manag. Care Pharm. 14 (6), 9–15. 10.18553/jmcp.2008.14.S6-B.9 18693783 PMC10438241

[B6] Bainbridge J. L. Ruscin J. M. (2009). Challenges of treatment adherence in older patients with Parkinson's disease. Drugs Aging 26 (2), 145–155. 10.2165/0002512-200926020-00006 19220071

[B7] Balkhi B. Almuaither A. Alqahtani S. (2023). Cross-national comparative study of orphan drug policies in Saudi Arabia, the United States, and the European Union. Saudi Pharm. J. 31 (9), 101738. 10.1016/j.jsps.2023.101738 37638213 PMC10458326

[B8] Bate R. Mathur A. Lever H. M. Thakur D. Graedon J. Cooperman T. (2016a). Generics substitution, bioequivalence standards, and international oversight: complex issues facing the FDA. Trends Pharmacol. Sci. 37 (3), 184–191. 10.1016/j.tips.2015.11.005 26687297

[B9] Bate R. Mathur A. Lever H. M. Thakur D. Graedon J. Cooperman T. (2016b). Generics substitution, bioequivalence standards, and international oversight: complex issues facing the FDA. Trends Pharmacol. Sci. 37 (3), 184–191. 10.1016/j.tips.2015.11.005 26687297

[B10] Baumgärtel C. (2012). Myths, questions, facts about generic drugs in the EU. Generics Biosimilars Initiative J. 1, 34–38. 10.5639/gabij.2012.0101.009

[B11] Besag F. M. (2000). Is generic prescribing acceptable in epilepsy? Drug Saf. 23 (3), 173–182. 10.2165/00002018-200023030-00001 11005701

[B12] Borgheini G. (2003). The bioequivalence and therapeutic efficacy of generic versus brand-name psychoactive drugs. Clin. Ther. 25 (6), 1578–1592. 10.1016/s0149-2918(03)80157-1 12860486

[B13] Bu H. Li Y. Jin C. Yu H. Wang X. Chen J. (2020). Overexpression of PRC1 indicates a poor prognosis in ovarian cancer. Int. J. Oncol. 56 (3), 685–696. 10.3892/ijo.2020.4959 31922238 PMC7010224

[B14] Carswell J. M. Gordon J. H. Popovsky E. Hale A. Brown R. S. (2013). Generic and brand-name L-thyroxine are not bioequivalent for children with severe congenital hypothyroidism. J. Clin. Endocrinol. Metabolism 98 (2), 610–617. 10.1210/jc.2012-3125 PMC3565118 23264396

[B15] Crawford P. Feely M. Guberman A. Kramer G. (2006). Are there potential problems with generic substitution of antiepileptic drugs? A review of issues. Seizure 15 (3), 165–176. 10.1016/j.seizure.2005.12.010 16504545

[B16] Davies G. (2001). Changing the salt, changing the drug. Pharm. J. 266.

[B17] de Barros C. M. Papoila A. L. (2007). Therapeutic profile of orphan medicines. Pharmacoepidemiol Drug Saf. 16 (4), 435–440. 10.1002/pds.1315 16958155

[B18] Department of Health and Social Care (2021). Community pharmacy drug reimbursement reform: consultation response. United Kingdom: DHSC.

[B19] Desmarais J. E. Beauclair L. Margolese H. C. (2011). Switching from brand-name to generic psychotropic medications: a literature review. CNS Neurosci. Ther. 17 (6), 750–760. 10.1111/j.1755-5949.2010.00210.x 21114789 PMC6493853

[B20] Di Paolo A. Arrigoni E. (2018). Generic substitution of orphan drugs for the treatment of rare diseases: exploring the potential challenges. drugs 78 (4), 399–410. 10.1007/s40265-018-0882-x 29464665

[B21] Drelichman G. Castañeda‐Hernández G. Ar M. C. Dragosky M. Garcia R. Lee H. (2020). The road to biosimilars in rare diseases‐ongoing lessons from Gaucher disease. Am. J. Hematol. 95 (3), 233–237. 10.1002/ajh.25701 31816110 PMC7027782

[B22] Elkoshi Z. Behr D. Mirimsky A. Tsvetkov I. Danon A. (2002). Multiple-dose studies can be a more sensitive assessment for bioequivalence than single-dose studies: the case with omeprazole. Clin. Drug Investig. 22 (9), 585–592. 10.2165/00044011-200222090-00003 29492852

[B23] Elmer S. Reddy D. S. (2022). Therapeutic basis of generic substitution of antiseizure medications. J. Pharmacol. Exp. Ther. 381 (2), 188–196. 10.1124/jpet.121.000994 35241634 PMC9132097

[B24] EMA (2019). Biosimilar medicines: marketing authorisation. Netherlands: European Medicines Agency. Available at: https://www.ema.europa.eu/en/human-regulatory-overview/marketing-authorisation/biosimilar-medicines-marketing-authorisation.

[B25] FDA (2015). Scientific considerations in demonstrating biosimilarity to a reference product: guidance for industry. Silver Spring: U.S. Department of Health and Human Services Food and Drug Administration. Available at: https://www.fda.gov/media/82647/download.

[B26] FDA (2024). Upcoming product-specific guidances for generic drug product development. US Food and Drug Administration. Available at: https://www.fda.gov/drugs/guidances-drugs/upcoming-product-specific-guidances-generic-drug-product-development.

[B27] Flather M. D. Farkouh M. E. Pogue J. M. Yusuf S. (1997). Strengths and limitations of meta-analysis: larger studies may be more reliable. Control. Clin. trials 18 (6), 568–579. 10.1016/s0197-2456(97)00024-x 9408719

[B28] Genazzani A. A. Pattarino F. (2008). Difficulties in the production of identical drug products from a pharmaceutical technology viewpoint. Drugs R. D. 9 (2), 65–72. 10.2165/00126839-200809020-00001 18298125

[B29] Gozzo L. Caraci F. Drago F. (2022). Bioequivalence, drugs with narrow therapeutic index and the phenomenon of biocreep: a critical analysis of the system for generic substitution. Healthcare 10 (8), 1392. 10.3390/healthcare10081392 35893214 PMC9394341

[B30] Guberman A. Corman C. (2000). Generic substitution for brand name antiepileptic drugs: a survey. Can. J. Neurol. Sci. 27 (1), 37–43. 10.1017/s0317167100051957 10676586

[B31] Håkonsen H. Eilertsen M. Borge H. Toverud E. L. (2009). Generic substitution: additional challenge for adherence in hypertensive patients? Curr. Med. Res. Opin. 25 (10), 2515–2521. 10.1185/03007990903192223 19708764

[B32] Heaney D. C. Sander J. W. (2007). Antiepileptic drugs: generic versus branded treatments. Lancet Neurol. 6 (5), 465–468. 10.1016/S1474-4422(07)70105-9 17434101

[B33] Hedges L. V. Olkin I. (2014). Statistical methods for meta-analysis. United States: Academic Press.

[B34] Hmingthansanga V. Singh N. Banerjee S. Manickam S. Velayutham R. Natesan S. (2022). Improved topical drug delivery: role of permeation enhancers and advanced approaches. Pharmaceutics 14 (12), 2818. 10.3390/pharmaceutics14122818 36559311 PMC9785322

[B35] Institute of Medicine Committee on Accelerating Rare Diseases R (2010). “Orphan product D the national academies collection: reports funded by national institutes of health,” in Rare diseases and orphan products: accelerating research and development. Editors Field M. J. Boat T. F. (Washington (DC): National Academies Press (US), National Academy of Sciences). 21796826

[B36] Jiang W. Makhlouf F. Schuirmann D. J. Zhang X. Zheng N. Conner D. (2015). A bioequivalence approach for generic narrow therapeutic index drugs: evaluation of the reference-scaled approach and variability comparison criterion. Aaps J. 17 (4), 891–901. 10.1208/s12248-015-9753-5 25840883 PMC4476992

[B37] Kearns G. L. Abdel-Rahman S. M. Alander S. W. Blowey D. L. Leeder J. S. Kauffman R. E. (2003). Developmental pharmacology--drug disposition, action, and therapy in infants and children. N. Engl. J. Med. 349 (12), 1157–1167. 10.1056/NEJMra035092 13679531

[B38] Loorand-Stiver L. Cowling T. Perras C. (2016). Drugs for rare diseases: evolving trends in regulatory and health technology assessment perspectives Ottawa: Canadian agency for drugs and technologies in health. Available at: https://www.cadth.ca/drugs-rare-diseases-evolving-trends-regulatory-and-health-technology-assessment-perspectives.

[B39] Meredith P. (2003). Bioequivalence and other unresolved issues in generic drug substitution. Clin. Ther. 25 (11), 2875–2890. 10.1016/s0149-2918(03)80340-5 14693311

[B40] Oza B. Radhakrishna S. Pipalava P. Jose V. (2019). Pharmacovigilance of biosimilars–Why is it different from generics and innovator biologics? J. Postgrad. Med. 65 (4), 227–232. 10.4103/jpgm.JPGM_109_19 31571620 PMC6813686

[B41] Sabatini S. Ferguson R. M. Helderman J. H. Hull A. R. Kirkpatrick B. S. Barr W. H. (1999). Drug substitution in transplantation: a national kidney foundation white paper. Am. J. Kidney Dis. 33 (2), 389–397. 10.1016/s0272-6386(99)70318-5 10023656

[B42] Sacks C. A. Van de Wiele V. L. Fulchino L. A. Patel L. Kesselheim A. S. Sarpatwari A. (2021). Assessment of variation in state regulation of generic drug and interchangeable biologic substitutions. JAMA Intern Med. 181 (1), 16–22. 10.1001/jamainternmed.2020.3588 32865564 PMC7489381

[B43] Saudi Food and Drug Authority (2023). Guidance for orphan drug designation. Saudi Food and Drug Authority. Available at: https://www.sfda.gov.sa/sites/default/files/2023-06/OrphanDrugDesignation.pdf.

[B44] Straka R. J. Keohane D. J. Liu L. Z. (2017). Potential clinical and economic impact of switching branded medications to generics. Am. J. Ther. 24 (3), e278–e289. 10.1097/MJT.0000000000000282 26099048 PMC5417581

[B45] Tassi R. A. Todeschini P. Siegel E. R. Calza S. Cappella P. Ardighieri L. (2017). FOXM1 expression is significantly associated with chemotherapy resistance and adverse prognosis in non-serous epithelial ovarian cancer patients. J. Exp. Clin. Cancer Res. 36, 63–18. 10.1186/s13046-017-0536-y 28482906 PMC5422964

[B46] Thiebaud P. Patel B. V. Nichol M. B. Berenbeim D. M. (2005). The effect of switching on compliance and persistence: the case of statin treatment. Am. J. Manag. Care 11 (11), 670–674. 16268750

[B47] Thorat C. Xu K. Freeman S. N. Bonnel R. A. Joseph F. Phillips M. I. (2012). What the Orphan Drug Act has done lately for children with rare diseases: a 10-year analysis. Pediatrics 129 (3), 516–521. 10.1542/peds.2011-1798 22371464

[B48] Tsang S.-F. Pandya S. Barakov K. Keutzer J. Lewis G. Ross L. (2022). Use of identical INN “imiglucerase” for different drug products: impact analysis of adverse events in a proprietary global safety database. Drug Saf. 45 (2), 127–136. 10.1007/s40264-021-01125-4 35020177 PMC8857131

[B49] Verbeeck R. K. Kanfer I. Walker R. B. (2006). Generic substitution: the use of medicinal products containing different salts and implications for safety and efficacy. Eur. J. Pharm. Sci. 28 (1-2), 1–6. 10.1016/j.ejps.2005.12.001 16413762

[B50] Williams A. C. Barry B. W. (2012). Penetration enhancers. Adv. drug Deliv. Rev. 64, 128–137. 10.1016/j.addr.2012.09.032 15019749

[B51] Xu E.-Y. Guo J. Xu Y. Li H.-Y. Seville P. C. (2014). Influence of excipients on spray-dried powders for inhalation. Powder Technol. 256, 217–223. 10.1016/j.powtec.2014.02.033

[B52] Yang G. Cintina I. Pariser A. Oehrlein E. Sullivan J. Kennedy A. (2022). The national economic burden of rare disease in the United States in 2019. Orphanet J. Rare Dis. 17 (1), 163. 10.1186/s13023-022-02299-5 35414039 PMC9004040

[B53] Zhang J. Wu K. Liu B. Hou S. Li X. Ye X. (2023). Bioequivalence study of ipratropium bromide inhalation aerosol using PBPK modelling. Front. Med. 10, 1056318. 10.3389/fmed.2023.1056318 PMC9941642 36824609

[B54] Zhao F. Siu M. K. Jiang L. Tam K. F. Ngan H. Y. Le X. F. (2014). Overexpression of forkhead box protein M1 (FOXM1) in ovarian cancer correlates with poor patient survival and contributes to paclitaxel resistance. PloS one 9 (11), e113478. 10.1371/journal.pone.0113478 25411964 PMC4239070

